# Courtship Sounds Advertise Species Identity and Male Quality in Sympatric *Pomatoschistus* spp. Gobies

**DOI:** 10.1371/journal.pone.0064620

**Published:** 2013-06-05

**Authors:** Silvia S. Pedroso, Iain Barber, Ola Svensson, Paulo J. Fonseca, Maria Clara P. Amorim

**Affiliations:** 1 Unidade de Investigação em Eco-Etologia, Instituto Superior de Psicologia Aplicada – Instituto Universitário, Lisbon, Portugal; 2 Department of Biology, College of Medicine, Biological Sciences and Psychology, University of Leicester, Leicester, United Kingdom; 3 Department Biology and Environmental Sciences, University of Gothenburg, Gothenburg, Sweden; 4 Departamento de Biologia Animal e Centro de Biologia Ambiental, Faculdade de Ciências da Universidade de Lisboa, Lisbon, Portugal; University of California Davis, United States of America

## Abstract

Acoustic signals can encode crucial information about species identity and individual quality. We recorded and compared male courtship drum sounds of the sand goby *Pomatoschistus minutus* and the painted goby *P. pictus* and examined if they can function in species recognition within sympatric populations. We also examined which acoustic features are related to male quality and the factors that affect female courtship in the sand goby, to determine whether vocalisations potentially play a role in mate assessment. Drums produced by the painted goby showed significantly higher dominant frequencies, higher sound pulse repetition rates and longer intervals between sounds than those of the sand goby. In the sand goby, male quality was predicted by visual and acoustic courtship signals. Regression analyses showed that sound amplitude was a good predictor of male length, whereas the duration of nest behaviour and active calling rate (i.e. excluding silent periods) were good predictors of male condition factor and fat reserves respectively. In addition, the level of female courtship was predicted by male nest behaviour. The results suggest that the frequency and temporal patterns of sounds can encode species identity, whereas sound amplitude and calling activity reflects male size and fat reserves. Visual courtship duration (nest-related behaviour) also seems relevant to mate choice, since it reflects male condition and is related to female courtship. Our work suggests that acoustic communication can contribute to mate choice in the sand goby group, and invites further study.

## Introduction

Acoustic signals convey crucial information on species, sex and individual identity as well as on individual motivation and quality [1]. As such, acoustic communication can provide a pre-zygotic isolating barrier and contribute to speciation in vocal taxa including birds [2], [3], anurans [4], [5] and insects [6], [7]. Variation in acoustic signals related to individual quality such as size, condition or other quality traits is also used in mate choice by different animals [8], [9], [10].

Among teleost fish, there is growing recognition of the role played by acoustic signals among the increasing number of species known to use acoustic communication to gain access to limited resources, such as food, territories or mates [11], [12], [13]. In comparison to tetrapods, fish have relatively simple central and peripheral vocal mechanisms and thus typically lack the ability to emit complex frequency-modulated calls [14]; for exception see [15], [16]. Consistent with this, comparative analyses of the acoustic signals of fishes has revealed that fine-scale temporal patterns – such as pulse number and pulse rate – are capable of encoding species identity ([17], [18], [19], [20], [21], but see [12]). In addition, calling rate, sound dominant frequency (the frequency with most acoustic energy), and the duration of pulses in a sound have been shown to advertise body size and condition [22], [23], [24], [25], while calling rate drives reproductive success in at least one fish species [13].

The relative simplicity of fish sounds and the possible lack of the confounding effects of learning [26], [27], make fish potentially useful models for studying the evolution of acoustic signalling, and for examining the extent to which sounds convey information relevant for species recognition and mate choice. Yet despite their utility, such studies are scarce in the literature. In this study we first aimed to compare acoustic mating signals in two congeneric, sympatric marine gobies – the sand goby (*Pomatoschistus minutus*) and the painted goby (*P. pictus*) – that exhibit similar life histories and breeding ecologies that overlap both spatially and temporally. Both species have a short life span, are polygamic and show exclusive paternal care [28]. Also, males of both species use low-frequency pulsed acoustic signals to lure the females into the nest for spawning [29], [30]. Second, we examined which acoustic features are related to male length and condition in the sand goby, which is an increasingly popular fish model in studies of sexual selection [31].

## Methods

### Ethics Statement

All experimental and animal care procedures comply with Swedish animal welfare laws, guidelines and policies and all efforts were made to maximize animal welfare. We caught fish with hand trawls from shallow bays near to the research station and immediately sorted them by species and gender into stock tanks, which were provided with a sand substrate and a continuous supply of fresh surface sea water. The sand goby males that produced sounds were euthanized with an excessive dose of anaesthetics (MS222, tricaine methane sulphonate; Pharmaq, Norway) and kept frozen (−80°C) until lipid quantification. Ethical permit 143–2011 from the Animal Ethics Committee of Gothenburg covered all experiments procedures reported here including fish collection.

### Study Species

The sand goby *Pomatoschistus minutus* and the painted goby *P. pictus* are small coastal species with a 1–2 years life span [28] that often live in sympatry. They typically breed for one season only, during which males and females spawn sequentially with different mates [32]. Mating occurs in nests that males build by excavating underneath bivalve shells that they also cover with sand. Females are then attracted for spawning with both conspicuous visual and acoustic signals [29], [30], (Video S1, S2). Parental care is provided solely by the male, who defends the nest from egg predators and tends the eggs until hatching [32].

**Figure 1:**
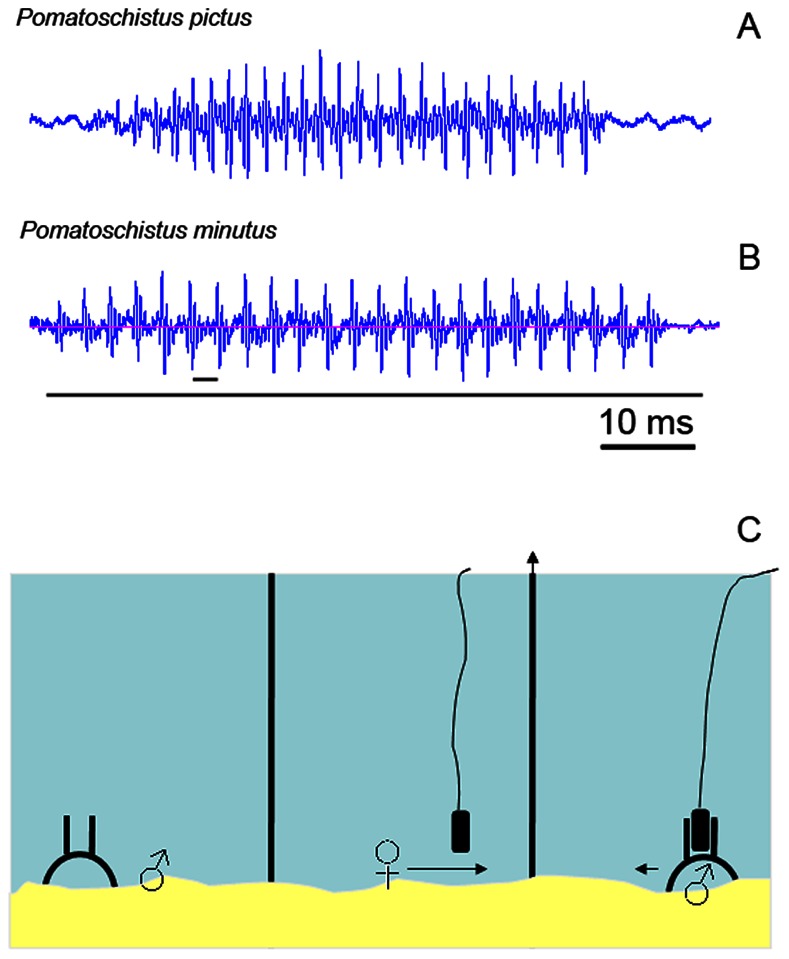
Courtship drums and recording setup. Oscillograms of courtship drums made by (a) the painted goby (*Pomatoschistus pictus*) and (b) the sand goby (*P. minutus*). The long line indicates sound duration whereas the short line depicts the interval between two consecutive pulses. (c) Setup used for the sand goby recordings illustrating the outer compartments occupied by territorial males and a female in the central compartment and the position of the two hydrophones. Trials began by removing one partition (illustrated by the arrow) which allowed interaction between one male and a female.

Breeding colouration and courtship differ slightly between these two species. Most sand goby males present a black rimmed anal fin, darkened tail, pelvic and dorsal fins, an iridescent blue band inside the black edge of the anal fin and a blue and black spot on the first dorsal fin [33]. Breeding painted goby males exhibit a darkened chin, black rimmed pelvic, anal and caudal fins, and the species-characteristic rows of dark spots of the first and second dorsal fins become more conspicuous than in non-breeding specimens. Most courtship interactions in the painted goby takes place outside the nest where males approach the female, make quiver displays, lead the female to the nest, nudge the female flank, and perform rapid eight swimming manoeuvres; but males also court the females from inside the nest with quivering displays [30]. Painted goby males produce two courtship acoustic signals: *drums*, which consist of low-frequency pulsed sounds (i.e. trains of pulses; Fig. 1a) associated with quivering displays and are made either outside or inside the nest, and short low-frequency non-pulsed *thumps*, which are associated with nest displays [30]. In the sand goby, courtship outside the nest mainly consists of males briefly approaching and displaying erected fins to the female before attempting to lead her to the nest. Sand goby males tend to spend longer periods at the nest, either remaining motionless with the head protruding at the nest entrance (Fig. S1). or in active nest displays, i.e. quivering and producing drum sounds [29]. Drums (Fig. 1b) are the only sound type in the sand goby, usually made from within the nest [29].

### Study Design

The study was conducted in May-June at the Sven Lovén Centre for Marine Sciences, at Fiskebäckskil on the Swedish west coast (58°15′N, 11°28′E). We caught fish with hand trawls from shallow bays near to the research station and sorted them by species and gender into stock tanks, which were provided with a sand substrate and a continuous supply of fresh sea surface water. The water fed to stock and experimental tanks was kept at a constant temperature (16°C) by a heat exchanger device. Fish were exposed to a natural photoperiod and fed daily *ad libitum* with chopped blue mussels.

We ran experiments in 26 l (painted goby) and 35 l (sand goby) aquaria divided in three compartments by transparent acrylic partitions. We placed a male in each lateral compartment with a shelter (Fig. 1c) and one gravid female centrally. We only used males that presented breeding colouration and constructed nests during an acclimatisation period, which lasted at least 24 h. Approximately 15 min before recordings we stopped water circulation and any noise source. We started trials by removing one partition, allowing one male to interact with the female for 20 min, while continuously recording all acoustic (see below) and visual (only in the sand goby, camcorder Sony DCR-SX65) behaviours. The video signal and a synchronized audio signal (derived from the audio recording chain) obtained in the sand goby trials were digitized with Pinnacle Dazzle DVD Recorder Plus (Pinnacle Systems, Mountain View, USA) to the laptop used for audio recordings. We used males (and females) in a maximum of two recording sessions to obtain enough sounds for analysis. After each experiment we removed the subject male and replaced the female. Males were weighed to the nearest 0.01 g (fresh weight, W) and measured to the nearest mm (standard length, SL). The sand goby males that produced sounds were euthanized with an excessive dose of anaesthetics (MS222, tricaine methane sulphonate; Pharmaq, Norway) and kept frozen (−80°C) until lipid quantification (see below). Silent sand goby males, all painted goby males and all females were returned to their natural habitat after trials.

Experimental aquaria used for the sand goby were insulated from room floor born noise by two layers of rubber foam shock absorbers located between the table and each of two wooden boards (1.5 cm thick). On top of these boards a styrofoam 4 cm thick layer supported the tanks. Sand goby male sounds were registered with a custom-made hydrophone [34] (frequency response within ±3 dB from 20 Hz to 1 kHz) and a High Tech 94 SSQ hydrophone (High Tech Inc., Gulfport, MS, USA; sensitivity −165 dB re1V/µPa; frequency response within ±1 dB from 30 Hz to 6 kHz), both connected to an A/D converter device (Edirol UA-25, Roland, Japan; 16 bit, 8 kHz) controlled by a laptop through Adobe Audition 3.0 (Adobe Systems Inc., Mountain View, CA, USA), allowing simultaneous two-channel recordings. The custom-made hydrophone was housed inside the male’s nest chimney while the other was placed in the central compartment. In the case of painted goby sound recordings, experimental aquaria were placed on top of a 7 cm thick styrofoam board and recordings were obtained only when external noise was minimal. Sounds were registered with a ITC hydrophone (ITC 8073, ITC, Santa Barbara, USA; sensitivity 167 dB re1V/µPa, frequency response within ±1.5 dB from 20 Hz to 2 kHz), suspended just above the male’s nest, connected to a Marantz PRC660 solid state recorder (Marantz, Eindhoven, Netherlands).

We analysed drum acoustic features with Raven 1.2.1 for Windows (Bioacoustics Research Program, Cornell Laboratory of Ornithology, Ithaca, NY, USA) following the methodology of [21] and [30]. We measured in both species sound duration (ms), number of pulses, pulse repetition rate (number of pulses divided for the sound duration, Hz), dominant frequency (the frequency where the sound has maximum energy, measured from power spectra: FFT size 8192 points, time overlap 60.0%, Hamming window, Hz) and drum interval (peak-to-peak interval between the last and the first pulses of two consecutive drums, ms). In the sand goby only we also measured sound pressure level (SPL, calibrated average RMS, dB re 1 µPa) from sounds registered in the nest where the hydrophone was fixed approximately 2 cm away from the male that keeps a rather stable position relative to the nest entrance. Note that SPL measurements should be taken as approximate values since not only recordings were carried out in the limited water body of the aquarium but also, and more importantly, any variation in the distance between the fish and the hydrophone can affect these measurements. The ratio between the average peak-to-peak interval of the 13^th^–16^th^ and the 3^rd^–6^th^ pulses was calculated to examine the existence of sound production fatigue in the sand goby (i.e. the decrease in pulse emission rate due to fatigue) and to check if the ability to sustain the rate of sound pulse emission depends on fish condition/quality. For the sand goby only, because sounds are emitted in rapid sequences (bursts), we further quantified drum burst duration (the duration of a sequence of drums, s; see [29] for details), burst interval (time interval between drum bursts, s; see [29] for details), as well as drum calling rate (drum min^−1^), active calling rate (considering only the min when fish vocalised, drum min^−1^) and calling effort (percentage of min spent calling). Drum calling rate, active calling rate and calling effort were calculated for the total 20 min of only one trial (the one with most acoustic/visual activity).

Twenty six painted goby males were tested for sound production. Of these only 16 out of 23 vocal males produced enough sounds to be included in the analysis. Vocal (analysed) painted goby males averaged 40 mm (± SD, range: ±2.8, 36–45 mm) in SL and 0.82 g (±0.15, 0.50–1.05 g) in W. We analysed an average of 67±38.3 (6–145) sounds for each painted goby male.

Thirty two sand goby males were tested from which 21 vocalised, and we analysed an average of 28±25.0 (6–108) sounds per vocal male. Vocal males averaged 44 mm in SL (±3.2, 39–49 mm) and 0.95 g (±0.22, 0.60–1.40 g) in W. Non-vocal sand goby males (N = 11) were similar sized to vocal males and averaged 46 mm (± SD, range: ±6.8, 38–45 mm) in SL and 1.14 g (±0.58, 0.70–1.10 g) in W.

We analysed sand goby behaviour from videos using EthoLog v.2.2 [35] and measured: duration of nest behaviour, i.e. resting with the head protruding from the nest entrance and nest quivering (s), total frequency of courtship outside the nest including approach, quiver outside the nest and lead (see above). We also quantified the latency for females to enter the male’s nest (s) and the frequency of female courtship behaviour, a characteristic bobbing movement exhibited by sexually receptive sand goby females in front of the male also accompanied by blackening of the eyes [33]. We only used one trial per male (as above, the one with most acoustic/visual activity) for behaviour analysis and only considered 10 min of video starting from the first male-female interaction.

### Lipid Quantification

We used both the condition factor (Fulton’s K, where K = W/SL^3^) [36] and body lipid content as metrics of male condition. Lipid content was measured in 21 sand goby males after [37]. In short, defrosted males were desiccated at 60°C for 24 h and weighed individually on a scale (Sartorius LE26P, Göttingen, Germany) to the nearest 0.001 mg. Lipids were extracted in 100 ml of petroleum ether (Sigma-Aldrich, St. Louis, USA) for 8 h. Body lipid content of each male was considered as the difference in dry weight before and after lipid extraction, and expressed as the lipid content relative to 100 g of fresh tissue.

### Data Analysis

We compared drum duration, number of pulses per drum, pulse repetition rate, dominant frequency and sound interval between species with *t* tests. As multiple statistical tests can increase type I errors (rejecting H_0_ when H_0_ is true) we chose an experimentwise error rate higher than the usual 5% (i.e. of 1%) in order to reduce the probability of type I errors while not compromising excessively statistical power, i.e. while avoiding increased chances of performing type II errors (not rejecting H_0_ when H_0_ is false) [13].

Drum interval was compared between species with a factorial ANOVA using sound interval classes as a factor with 7 levels (5 classes of 100 ms below 0.5 s, plus 0.5–1 s and >1 s), and species as a factor with two levels. We quantified the percentage of occurrences in each sound interval class and square root arcsin-transformed the data to meet the ANOVA assumptions, as percentages typically follow a binomial distribution [38]. As acoustic features were not related with male SL in either species (Pearson correlation, *P. pictus*: N = 15, R = −0.32–0.10, *P*>0.05; *P. minutus*: N = 21, R = −0.37–0.39, *P*>0.05) we did not control comparisons between species for male size.

We used multiple linear regression analyses (Table 1) to determine if acoustic and visual behavioural variables were good predictors of male quality using a stepwise selection procedure (*P*≤0.05 to add and *P*≥0.10 to remove). We used male SL, K condition factor and relative lipid content as dependent variables in three regression models. We included as possible predictors the male mean values for both acoustic and visual behaviour parameters. We considered all acoustic parameters plus nest behaviour duration (nest quiver and rest) and total number of courtship events performed outside the nest. We used log_10_-transformation [38] to normalise male SL and the predictors drum duration, number of pulses, sound burst duration, burst interval, drum rate, calling effort, nest behaviour and courtship outside the nest.

**Table 1:** Dependent variables and predictor used in the linear regression models.

Dependent variables	Predictors
male SL* or	***Acoustic variables***
male K condition factor or	drum duration* (ms)
male K condition factor	number of pulses*
	pulse repetition rate (Hz)
	dominant frequency (Hz)
	sound pressure level (SPL, dB re 1 µPa)
	sound production fatigue
	drum interval (ms)
	drum burst duration* (s)
	burst interval* (s)
	drum calling rate* (drum min−^1^)
	active calling rate (drum min−^1^)
	calling effort*
	***Visual behaviour variables***
	nest behaviour duration (nest quiver and rest)*
	total no. of courtship events performed outside the nest*
Number of female courtship events*	all acoustic variables (as above)
	all visual behaviour variables (as above)
	male SL (mm)
	male K condition factor
	male relative lipid content (g)

* Data were log_10_-transformed to meet the linear regression model assumptions. Abbreviations and units are shown between brackets.

We further tested which factors influenced female courtship behaviour, i.e. female courtship and latency to enter the nest, using linear regression analysis (Table 1). As female courtship was correlated with latency to enter the nest (Pearson correlation, R = 0.59, *P* = 0.005) we only carried out this analysis using female courtship as a dependent variable (log_10_-transformed). We used as predictors all the acoustic and visual behaviour variables as above and male quality features, SL, K and lipids. We further correlated female courtship with female SL and K condition factor (measured before trials) to ascertain if female size and K (a proxy for roundness) are related to female courtship behaviour.

All final regression models complied with all assumptions of multiple linear regression. All model residuals were normally distributed. We assessed autocorrelation of residuals and multicollinearity of predictors with Durbin–Watson statistics and with the variance inflation factor (VIF). We performed further residual analysis by visually inspecting residual plots.

We conducted statistical analyses with SPSS for Windows (20.0, SPSS Inc., New York, USA) and Statistica (10, Statsoft Inc., Tulsa, USA).

## Results

### Differences between Species

**Figure 2:**
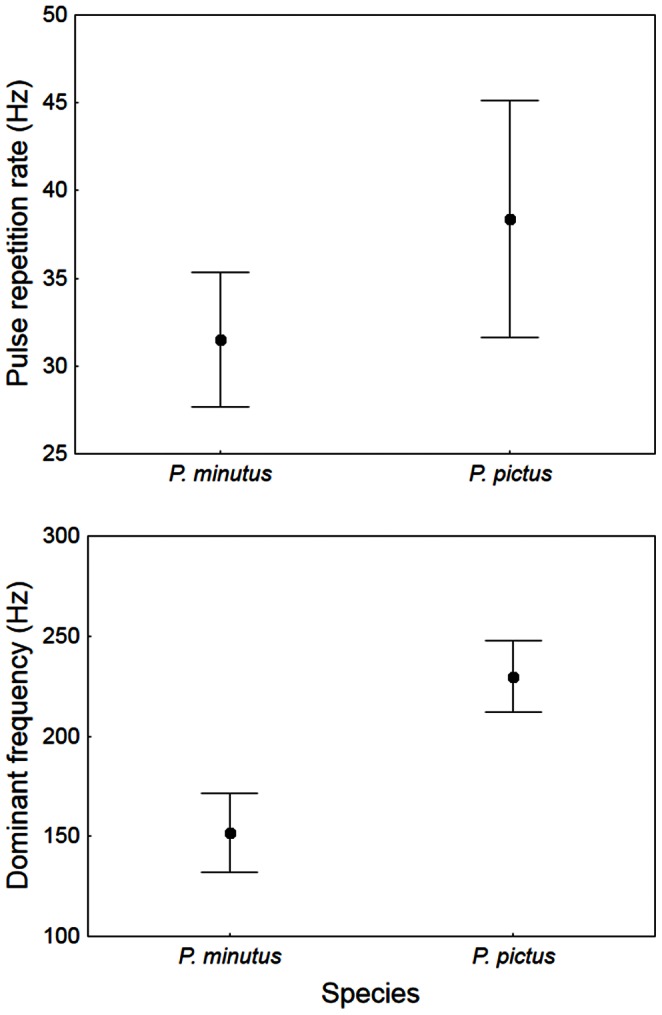
Comparison of courtship drums produced by males of *Pomatoschistus minutus* and *P. pictus*. Symbols show means for pulse repetition rate and dominant frequency and error bars represent ±1 standard deviation.

**Figure 3:**
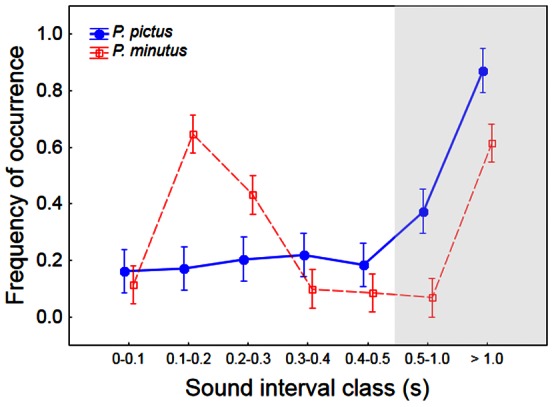
Frequency of occurrence of time intervals between consecutive drums in *P. minutus* and *P. pictus*. Drum intervals were square root arcsin-transformed. Note that in the shaded area broader interval classes than the ones represented on the left area of the graph are shown. Symbols show means and error bars depict 95% confidence intervals.

Both sand and painted gobies made low frequency drums (main frequencies below 300 Hz) lasting less than 1 s and composed of *c*. 24 pulses (Fig. 1; Table 2). Drums did not differ significantly between species in sound duration (*t* test, *t* = 1.75, *df* = 28.8, *P*>0.05) or number of pulses (*t* = 0.43, *df* = 35, *P*>0.05) (Table 2). Both pulse repetition rate and dominant frequency were significantly higher in the painted than in the sand goby (pulse repetition rate: *t* = −3.92, *df* = 35, *P*<0.001; dominant frequency: *t* = −12.26, *df* = 35, *P*<0.001; Fig. 2). A comparison of sound intervals between species using sound interval class and the species as factors showed that, although there were no significant differences between species in the mean sound interval, the sand goby produced sounds with intervals between 0.1 and 0.3 s significantly more frequently than the painted goby whereas the painted goby drummed with intervals larger than 0.5 s more often than the sand goby (ANOVA, interaction *F*_5,210_ = 1.33, *P*<0.001; sound interval class *F*_5,210_ = 0.80, *P*<0.001; species *F*_1,210_ = 0.11, *P*>0.05; Fig. 3).

**Table 2:** Acoustic features of drums produced during courtship by males of *Pomatoschistus pictus* and *P. minutus*.

	*P. pictus*				*P. minutus*			
Acoustic parameters	Mean	SD	Range	Range_abs_	Mean	SD	Range	Range_abs_
Drum duration (ms)	628.8	171.8	358.0–1118.8	29–4288	797.2	395.4	387.4–1914.1	108–8044
No. of pulses	23.3	6.5	11.2–38.0	2–139	24.8	12.1	12.2–62.7	3–229
Pulse repetition rate (Hz)	38.4	6.7	27.2–52.1	19.5–64.7	31.5	3.8	25.0–39.3	15.2–47.6
Dominant frequency (Hz)	229.8	18.0	194.2–255.0	144–266	151.9	19.6	126.7–187.9	94.7–236
Drum interval (s)	2.27	0.74	1.24–3.29	–	0.95	0.69	0.16–2.90	0.06–0.63
Number of sounds in a burst	–	–	–	–	3.5	1.58	1.1–7.0	1–12
Drum burst duration (s)	–	–	–	–	2.5	0.98	0.98–4.85	0.14–9.75
Burst interval (s)	–	–	–	–	81.3	71.14	5.8–289.4	0.5–867.3
Drum calling rate(drum min^−1^)	–	–	–	–	1.7	1.36	0.2–4.3	–
Active calling rate(drum min^−1^)	–	–	–	–	7.4	3.64	1.9–15.5	–
Calling effort	–	–	–	–	16.2	10.12	6.9–42.9	–
Sound pressure level(dB re 1 µPa)	–	–	–	–	90.7	5.11	83.8–98.1	78.5–137.3
Sound production fatigue	–	–	–	–	1.1	0.09	0.9–1.3	0.8–1.9

Descriptive statistics is based on male means except for absolute range values (range_abs_) that concern all data (*P. pictus*: 1073 drums and 884 sound intervals from 16 males; *P. minutus*: 580 drums and 594 sound intervals from 21 males). Sound intervals only concern interval up to 10 s.

### Predictors of Size and Condition in the Sand Goby

Vocal males ranged in size between 39–49 mm SL (mean ± SD = 43.6±3.2 mm, cv = 0.07), in condition factor between 0.9–1.4 (1.1±0.15, cv = 0.13) and showed a large individual variability in relative lipid content: 1.1–3.9 g (2.3±0.75 g, cv = 0.32). Likewise, males varied extensively in their levels of acoustic (Table 2) and visual courtship activity. Calling rate varied between 0.2–4.3 drum min^−1^ (1.7±1.36 drum min^−1^, cv = 0.79) and active calling rate, that accounted only for the time spent calling, ranged between 1.9–15.5 drum min^−1^ (7.4±3.64 drum min^−1^, cv = 0.49). Nest behaviour (quiver and rest) lasted from 0–533.7 s (131.3±173.7 s, cv = 1.32) whereas males performed between 0–11 courtship displays outside the nest (4.5±3.37, cv = 0.75). All but one vocal male (i.e. 20 males) succeeded in attracting the female into the nest and only 3 vocal males did not receive any eggs. Silent males were not successful in obtaining eggs in the nest.

**Figure 4:**
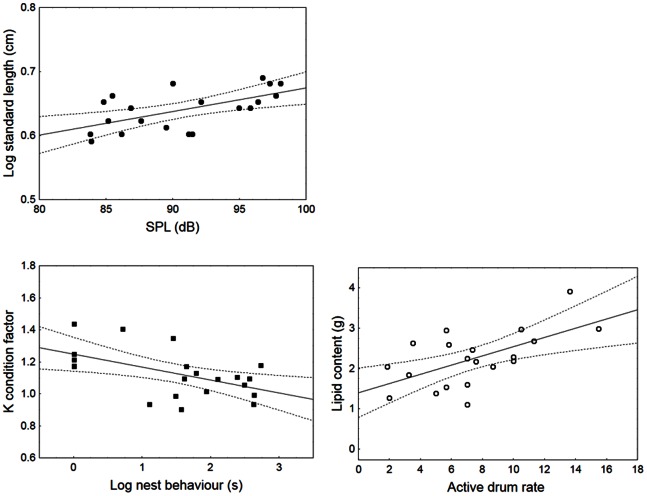
Relation between visual and acoustic predictor variables and male quality parameters in *P. minutus*. Predictors included in the final linear regression models were male nest behaviour duration, sound pressure level (SPL) and active drum rate (drums min^−1^). Regression lines and 95% confidence interval bands are shown.

The best regression model showed that only drum SPL was a good predictor of male length with louder males having a higher SL. Drum SPL accounted for 36% of the variation in body length (Table 3; Fig. 4). Male condition variables were significantly predicted by male visual and acoustic behaviour, with males exhibiting shorter periods of nest behaviour and higher calling rates having a higher K condition factor and relative higher lipid levels respectively. Variation in nest behaviour (quiver display and rest) significantly explained 29% of the observed variability in K condition factor and active calling rate significantly explained 38% of lipid content variability (Table 3; Fig. 4). Our model predicts that males differing in active calling rate by 10 drums min^−1^ (e.g. 12 vs. 2 drum min^−1^) would indicate a difference of 1.2 g in their relative lipid content.

**Table 3:** Table for predictors of male sand goby *P. minutus* standard length (SL) and condition (K condition factor and body lipid content).

Dependent variable	Includedpredictor	*B*	S.E.M.	*t*	*P*	*r*	*F*	Model significance	R^2^	DW	VIF
SL	Intercept	0.31	0.11	2.92	0.009						
	SPL	0.004	0.01	3.16	0.005	0.60	*F*_1,19_ = 10.00	*P* = 0.005	0.36	2.75	1.0
K	Intercept	1.25	0.05	24.00	<0.001						
	Nest	−0.08	0.03	−2.70	0.015	−0.54	*F*_1,19_ = 7.31	*P* = 0.015	0.29	2.2	1.0
Lipid	Intercept	1.37	0.29	4.75	<0.001						
	Active drum rate	0.18	0.04	3.34	0.004	0.62	*F*_1,19_ = 11.17	*P* = 0.004	0.38	1.9	1.0

Nest behaviour duration (quiver and rest) and SL were log_10_-transformed to meet the linear regression model assumptions. r – partial correlation between the dependent variable and the predictor, controlling for the effects of the other predictors in the model. DW - Durbin Watson statistics.

### Factors Influencing Female sand Goby Courtship Behaviour

**Figure 5:**
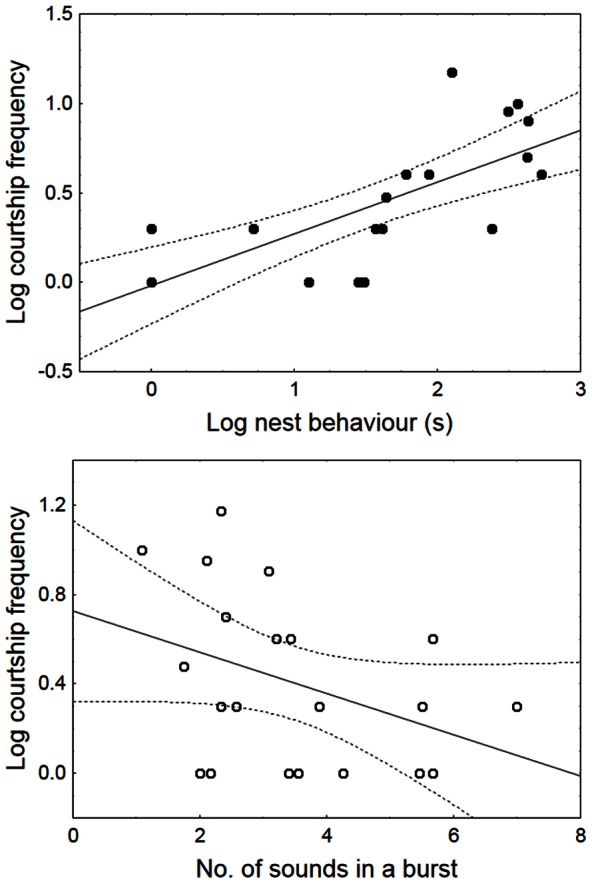
Relation between male courtship behaviour and the frequency of female courtship in *P. minutus*. Male courtship behaviour predictors in the final linear regression model were nest behaviour duration and the number of sounds in a burst. Regression lines and 95% confidence interval bands are shown.

Females took between 13.1–600 s (165.6±138.4 s, cv = 0.84) to enter the males’ nest and one female did not enter the nest (latency = 600 s) although she courted the male four times and the male performed nest behaviour for 421 s from which 55% was spent actively courting the female, i.e. quivering. Fourteen out of 21 females courted the males. Female courtship events ranged from 0–14 (2.8±3.8, cv = 1.36), with 86% of the variation in the number of female courtship events being explained by male nest behaviour (quiver and rest) and vocal activity. Nest behaviour alone explained 78% of the observed variation in female courtship and the number of drums in a burst further explained 8% of data variability (Table 4). Nest behaviour was positively related to female courtship whereas the number of sounds in a burst correlated negatively (Fig. 5). Female courtship was not related with female size (Pearson correlation, N = 20, R = −0.23, *P*>0.05) or K condition factor before mating (R = −0.18, *P*>0.05).

**Table 4:** Table for predictors of female sand goby *P. minutus* courtship.

Dependentvariable	Includedpredictor	*B*	S.E.M.	*t*	*P*	*r*	*F*	Modelsignificance	R^2^	DW	VIF
Female courtship	Intercept	0.26	0.12	2.19	0.04						
	Nest	0.27	0.04	6.43	<0.001	0.84					1.0
	Sounds in burst	−0.08	0.03	−3.01	0.008	−0.59	*F*_2,19_ = 24.75	*P*<0.001	0.86	2.1	1.0

Nest behaviour duration (quiver and rest) and female courtship were log_10_-transformed to meet the linear regression model assumptions. r – partial correlation between the dependent variable and the predictor, controlling for the effects of the other predictors in the model. DW - Durbin Watson statistics.

## Discussion

Species recognition and mate choice critically important in animal communication and in making the ‘correct’ choices often relies on the female’s response to variation in male traits [39]. Yet there are few studies in the literature that simultaneously explore female mate recognition and quality evaluation in the same fish species [18], [20]. Here we show that the simple drums produced by male sand gobies while courting the females potentially contain important information in both species recognition and mate assessment. We also show that male visual behaviour influences female courtship, exhibited by sexually receptive sand goby females [33].

### Inter-specific Differences

Both sand and painted goby males produce low frequency drumming sounds, mainly while quivering in the nest, to entice the females to enter the nest and spawn (present study and [29], [40]). Drums produced by the painted goby showed significantly higher dominant frequencies than those produced by the sand goby. Although higher dominant frequencies are expected in smaller individuals of some fishes [24], [41], [42] we did not observe a relationship between this acoustic parameter and fish length in either studied species. Also, fish SL overlapped considerably in both species (36–45 mm SL in the painted goby vs. 39–49 mm SL in the sand goby) suggesting that size alone is not responsible for inter-specific differences in sound frequency. Malavasi and colleagues [21] have studied five different genera of vocalising gobies from the Mediterranean, including *Pomatoschistus,* and consistently showed a significant relationship between body size and dominant frequency only in the grass goby *Zosterissessor ophiocephalus.* Sound frequency could hence provide a reliable cue for species recognition in *Pomatoschistus* spp. since it appears to be independent of body size and falls well within their hearing sensitivity and expected frequency discriminating abilities [43], [44].

The present study also revealed that the pulse repetition rate and sound intervals differed significantly between the two studied species, with the painted goby showing higher pulse rates within a sound and longer intervals between sounds. Although few systematic comparisons of acoustic signals are available for closely-related fish species [21], temporal characteristics of sounds are thought to be important carriers of species-specific information in fish. In fact distinct temporal patterns, such as pulse number and rate, often differentiate sounds of closely-related sympatric species such as in the Pomacentridae, Cichlidae and Mormyridae [18], [20], [41], [45]. Further, playback experiments testing male pomacentrids from the genus *Stegastes* provided evidence that fish can distinguish conspecific courtship sounds from those of closely-related congenerics, based on the number of pulses and pulse rate [17], [45]. Our results are consistent with the hypothesis that the pulse rate – and possibly also the pattern of sound emission (i.e. the sequence considering sound intervals) – contribute to the recognition of conspecific mates, although sound intervals show higher intra-specific variability than pulse rate (Table 2) and thus potentially provide less reliable species-specific acoustic cues.

### Acoustic and Visual Cues for Mate Choice

Sand goby males examined in the study presented a relatively small variability (CV) in male length and K condition factor (<15%) but showed considerable variability in their fat reserves (32%). Further, acoustic and visual courtship behaviour was also highly variable among males (75–132%) providing a basis for mate assessment. All male quality parameters (SL, K and lipids) were predicted by male visual or acoustic features. Sound amplitude significantly explained 36% of the variation in male length. According to the best multiple regression model, a variation of 1 cm in SL (e.g. 4–5 cm) corresponds to a change of 24 dB in sound amplitude (see Table 3 and Fig. 4), comparable to the findings of [29] for Baltic sea populations of sand goby and of [40] for the painted goby. Male size is important for the sand goby as larger males have a competitive advantage over nest sites [46], [47] and are preferred by females [48]. If females of the studied sand goby population are selecting larger mates to increase their fitness, then they may also use sound amplitude as a redundant signal of male quality. In insects, anurans and birds, variation in male sound amplitude plays an important role in both female choice and male-male competition, and females of several species have been found to prefer louder calls [9], [49], [50].

We also found that male sand goby condition – an important determinant of competitive dominance over nests sites when body size differences are small [51] – could be predicted by male behaviour. Indeed, 29% of variability in male K condition factor and 38% of the variability in male fat reserves could be explained by nest behaviour duration and active calling rate, respectively. In general, courtship behaviour is likely to impose energy costs [52], [53]. However, the energetic costs of visual courtship in the sand goby seem controversial [37], [54], [55], and in the painted goby visual courtship does not relate to the male condition factor or fat reserves [40]. On the other hand, calling activity in fish with parental care appears to advertise male condition such as fat reserves (sand goby - present study; Lusitanian toadfish [25]; painted goby [40]) that are key to prolonged nest defence and general parental activities [56]. In these cases, calling activity is likely under mate choice since males that vocalize more frequently enjoy a higher reproductive success [13], [40]. Interestingly, both in the sand goby (present study) and in the painted goby [40], a relationship between fat reserves and calling activity could be detected even over a short (20 min) observation period, suggesting that only males in good condition have the ability to pay the costs of intense calling bouts. In birds, in which the sexual function of song has been well established, male song rate may also influence reproductive success [57], [58].

### Female Sand Goby Courtship Behaviour

Female courtship was positively related with the duration of male nest behaviour, which explained most of the variability of this female behaviour (78%). Female courtship has been documented as a signal of sexual receptivity in the sand goby [33]. In the present study, however, all but one of the females that did not court spawned in the male’s nest, indicating that they were also sexually receptive. It is possible that the relative quality of mates may have affected courtship and assessment time leading to variability in mutual courtship, i.e. male nest behaviour and female courtship. Females could be assessing male condition through nest behaviour duration since it appears to be a good predictor of male condition factor. On the other hand, males could also be assessing female quality including fecundity. It is becoming clear that preference for mates may vary between and within individuals and that the perception of variability in mate- or self-quality can influence mating preferences and the mate assessment process [59], [60]. However this hypothesis still needs to be tested.

Gobies from the genus *Pomatochistus* are very similar morphologically [61] and use the same sensory channels during mate attraction [21] facing the potential costs of wasting time, energy, nutrients, or gametes in erroneous heterospecific sexual interactions [62] that may lead to fitness loss associated with reproductive interference, i.e. the process of mate acquisition that adversely affects the fitness of at least one of the species involved and that is caused by incomplete species recognition (reviewed in [63]). Reproductive interference might cause from only a slight decrease in fitness to the displacement of one species. Hence, traits that reduce these costs should be positively selected and lead to reinforcement of premating barriers, such as character displacement, or promote ecological segregation of species [64]. We have shown that dominant sound frequency and temporal patterns of vocal signals may potentially enable species recognition in two sympatric *Pomatoschistus* species, consistent with other empirical studies on sympatric closely-related fish species. In turn, sound amplitude and active calling rate may contribute to conspecific mate assessment and preference within the sand goby. Future studies should test if these or other acoustic features are in fact used in species recognition and in mate choice in fish. We propose the sand goby as a major model species to address these fairly unexplored questions. In particular the use of acoustic signals in conspecific vs. heterospecific mate recognition still remains to be demonstrated in fish.

## Supporting Information

Figure S1 ***Pomatoschistus minutus*** **in experimental nest.** Note the nest chimney that houses the hydrophone.
(JPG)

Video S1 **Courtship behaviour in the painted goby** ***Pomatoschistus pictus*** **.** The male leads the female into the nest and makes drumming sounds before and after she enters the nest.
(MP4)

Video S2 **Courtship behaviour in the sand goby** ***Pomatoschistus minutus*** **.** The male leads the female into the nest and drums while she is outside the nest. The female approaches with blackened eyes (a signal of spawning readiness) and the male nudges the female flank. The male continues to drum after the female enters the nest.
(MP4)
