# Supplementary figures and images for: Courtship Sounds Advertise Species Identity and Male Quality in Sympatric Pomatoschistus spp. Gobies
Source: PLoS One. 2013 Jun 5;8(6):e64620. doi: 10.1371/journal.pone.0064620 (PMC3674009; doi:10.1371/journal.pone.0064620)

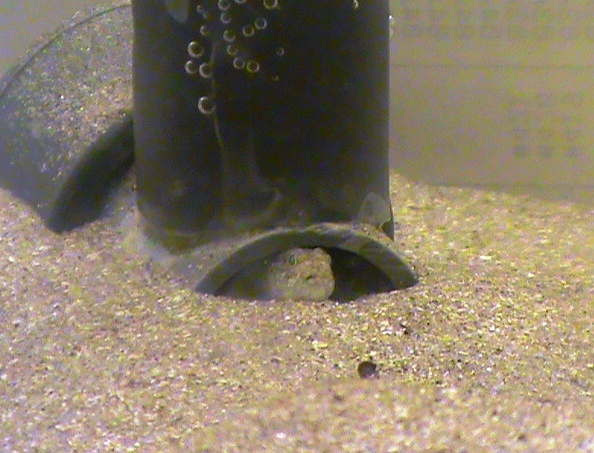

Supplement: Figure S1 — Pomatoschistus minutus in experimental nest. Note the nest chimney that houses the hydrophone. (JPG) [file pone.0064620.s001.jpg]
